# Global gene expression profiles of canine macrophages and canine mammary cancer cells grown as a co-culture *in vitro*

**DOI:** 10.1186/1746-6148-8-16

**Published:** 2012-02-21

**Authors:** Magdalena Król, Karol M Pawłowski, Kinga Majchrzak, Małgorzata Gajewska, Alicja Majewska, Tomasz Motyl

**Affiliations:** 1 Department of Physiological Sciences, Faculty of Veterinary Medicine, Warsaw University of Life Sciences - WULS, Nowoursynowska 159, 02-776 Warsaw, Poland; 2 Department of Animal Environment Biology, Faculty of Animal Sciences, Warsaw University of Life Sciences - WULS, Ciszewskiego 8, 02-786 Warsaw, Poland

## Abstract

**Background:**

Solid tumours comprise various cells, including cancer cells, resident stromal cells, migratory haemopoietic cells and other. These cells regulate tumour growth and metastasis. Macrophages constitute probably the most important element of all interactions within the tumour microenvironment. However, the molecular mechanism, that guides tumour environment, still remains unknown. Exploring the underlying molecular mechanisms that orchestrate these phenomena has been the aim of our study.

A co-culture of canine mammary cancer cells and macrophages was established and maintained for 72 hrs. Having sorted the cells, gene expression in cancer cells and macrophages, using DNA microarrays, was examined. The results were confirmed using real-time qPCR and confocal microscopy. Moreover, their ability for migration and invasion has been assessed.

**Results:**

Microarray analysis showed that the up-regulated genes in the cancer cell lines are involved in 15 highly over-manifested pathways. The pathways that drew our diligent attention included: the inflammation pathway mediated by chemokine and cytokine, the Toll receptor signalling pathway and the B cell activation. The up-regulated genes in the macrophages were involved in only 18 significantly over-manifested pathways: the angiogenesis, the p53 pathway feedback loops2 and the Wnt signalling pathway. The microarray analysis revealed that co-culturing of cancer cells with macrophages initiated the myeloid-specific antigen expression in cancer cells, as well as cytokine/chemokine genes expression. This finding was confirmed at mRNA and protein level. Moreover, we showed that macrophages increase cancer migration and invasion.

**Conclusions:**

The presence of macrophages in the cancer environment induces acquisition of the macrophage phenotype (specific antigens and chemokines/cytokines expression) in cancer cells. We presumed that cancer cells also acquire other myeloid features, such as: capabilities of cell rolling, spreading, migration and matrix invasion (what has also been confirmed by our results). It may, perhaps, be the result of myeloid-cancer cell hybrid formation, or cancer cells mimicking macrophages phenotype, owing to various proteins secreted by macrophages.

## Background

Solid tumours comprise cancer cells, resident stromal cells, and migratory haemopoietic cells. Intricate interactions between the cell types regulate tumour growth, progression, metastasis, and angiogenesis. Macrophages are an important element of these microenvironment interactions [1]. They may represent either M1 or M2 phenotype. The classical activation by microbial products is that of the M1 phenotype (also thought to have anti-tumour properties), whereas alternative activation (caused by cancer cells) drives macrophages conversion toward the M2 phenotype. Cancer cells are known to release various chemoattractants which recruit macrophages to colonize the tumour site [2]. On the other hand, counter-activated tumour-associated macrophages (TAMs) produce chemokines, cytokines, growth and angiogenic factors [1,3], thus they actively contribute to tumour progression and their transition to malignancy. Exploring the underlying molecular mechanisms of this phenomenon seems to be utterly important. Therefore, to view and explain the molecular interactions between the cancer cells and TAMs, we established an *in vitro *co-culture and conducted a global gene expression analysis using DNA microarrays of macrophages and cancer cells. Neither there is an abundance of microarray data on the global gene expression in TAMs [2,4,5] available, nor there is much information on the changes of cancer cells and their gene expression whilst cultured with macrophages.

The findings confirm that cancer cells under co-culture conditions acquired the macrophage-specific antigen expression. It could as well be indicative of these cells also having other phenotypic characteristics of macrophages, such as: capabilities of cell rolling, spreading, diapedesis, or migration, that allow the metastasis process. Our *in vitro *studies confirmed that macrophages enhance tumour migration and invasion.

## Methods

### Cell lines

The cell lines used for the study have previously been used in other published research [6-9]. Two canine mammary adenocarcinoma cell lines (CMT-W1, CMT-W2), anaplastic cancer cell line (P114), simple carcinoma cell line (CMT-U27) and spindle-cell mammary tumor cell line (CMT-U309) were examined. CMT-W1 and CMT-W2 cell lines were kindly donated by Prof. Dr. Maciej Ugorski and Dr. Joanna Polanska from Wroclaw University (Poland), CMT-U27 cell line was kindly donated by Dr. Eva Hellmen from Swedish Agricultural University (Sweden) and P114 cell line was kindly donated by Dr. Gerard Rutteman from Utrecht University (The Netherlands).

Cells were cultured under optimal conditions: a medium RPMI-1640 enriched with 10% (v/v) heat-inactivated fetal bovine serum (FBS), penicillin-streptomycin (50 iU mL-1), and fungizone (2.5 mg mL-1) (reagents obtained from Sigma Aldrich, USA), in an atmosphere of 5% CO2 and 95% humidified air at 37°C, and routinely subcultured every other day.

### Canine blood mononuclear cell separation

The anticoagulated whole blood from healthy dogs (patients of the Department of Small Animal Diseases with Clinic, Faculty of Veterinary Medicine, Warsaw University of Life Sciences, Poland) was collected for routine diagnostic purposes (in that case the ethical committee permission is not required). The remaining volume of the blood samples was taken to our analyses (with the written permission of the dog's owners) and immediately subjected to a mononuclear cell separation using Accuspin System-Histopaque 1077 (Sigma Aldrich, USA) according to the manufacturer's protocol. The blood specimen was placed on a porous high-density polyethylene barrier, separating lower chamber containing the Histopaque-1077 solution, in a sterile centrifuge tube. The tube was centrifuged at 800 × *g *for 30 min at room temperature. On centrifugation, erythrocytes and granulocytes descend through the frit to pellet below the Histopaque-1077. Lymphocytes and monocytes remained above the frit on the plasma-Histopaque-1077 interface. This layer of cells was aseptically removed with a pipette and transferred to a sterile 15-ml centrifuge tube. Then, the cells were washed with PBS once and subjected to further procedures.

### Monocyte sorting and culturing

**Figure 1:**
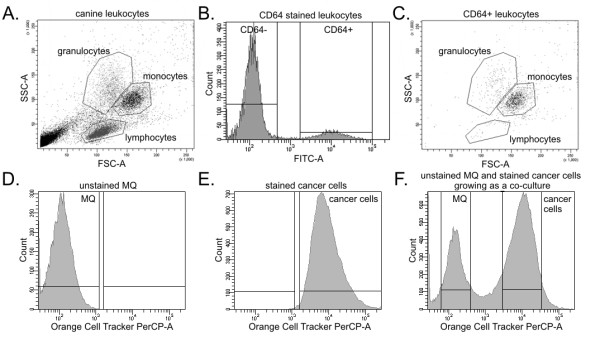
**FACS mononuclear blood cells and co-culture analysis and sorting**. Cytograms and histograms obtained using FACS Aria II (Becton Dickinson, USA). (**A**) The cytogram of mononuclear cells obtained from canine blood after the Accuspin System-Histopaque 1077 centrifugation. The monocytes were gated based on the morphological criteria (SSC v/s FSC cytogram). (**B**) Histogram of CD64 stained leukocytes: CD64 negative (CD64-) leukocytes showed low FITC signal, whereas CD64 positive (CD64+) leukocytes showed high FITC signal. (**C**) The cytogram of CD64-positive cells showing antibodies specificity to monocytes. (**D**) Histogram of the fluorescence intensity of unstained macrophages grown as a single culture. (**E**) Histogram of the fluorescence intensity of CMTMR-stained cancer cells grown as the single culture. (**F**) Histogram and sorting gates of unstained macrophages and CMTMR-stained cancer cells grown as a co-culture for 72 hrs.

The isolated mononuclear blood cells were incubated for an hour at room temperature with mouse monoclonal anti-CD64 FITC-conjugated (Becton Dickinson, USA) antibodies (specific for monocytes/macrophages and minimally specific for granulocytes) applied in a volume recommended by the manufacturer of 20 μl per 10^6 ^cells suspended in 100 μl. The leukocytes were analyzed using FACS Aria II (Becton Dickinson, USA). The monocytes were at first identified and gated based on the morphological criteria (SSC v/s FSC cytogram) as an intermediate in size (FSC) and with an intricate nuclear configuration (intermediate SSC) cells (Figure 1A). The cytogram showed that most of the granulocytes were eliminated during the mononuclear cells isolation. Then, the CD64-positive cells were gated (Figure 1B) and specificity of staining was checked, showing only the CD64-positive cells on the cytogram (Figure 1C).

The CD64-positive monocytes were then separated and grown as a co-culture with cancer cells, as well as a mono-culture. The culturing conditions were the same as those for cancer cells. According to the subject data, culturing of monocytes for 72 hrs is sufficient for their differentiation towards macrophages [10,11].

### Co-culture

In this model, cancer cells (CMT-W1, CMT-W2, CMT-U27, CMT-U309, P114) were grown on separated flasks, and sorted monocytes were then layered on the top of each cell line. An Orange CellTracker fluorescent dye CMTMR (Invitrogen, USA) was used to stain the cancer cells population before the sorted monocyte population was added. Staining was accomplished by incubation in serum/antibiotics-free RPMI medium containing 5 μM CMTMR (10 mM stock in DMSO; Sigma Aldrich, USA) for 45 min at 37°C. Subsequently, the medium was aspirated, and the cancer cells were washed twice with PBS and incubated with complete RPMI for 1 hr. Sorted monocytes were placed on the CMTMR-stained cancer cells.

**Figure 2:**
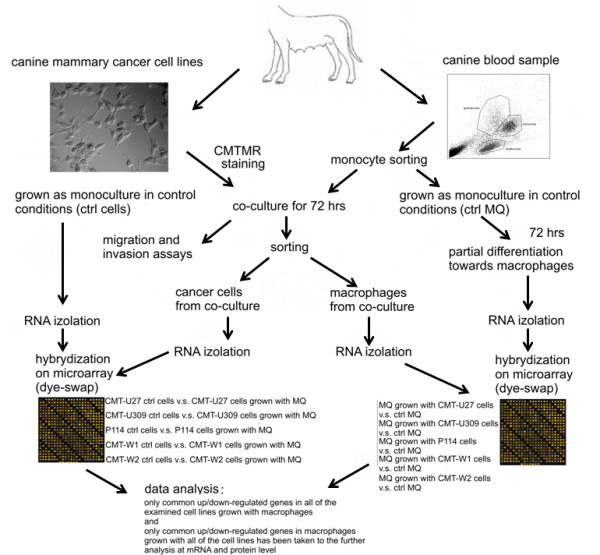
**Plan of the experiments**. The scheme of the experiments conducted within hereby study.

The co-culture was maintained for at least 72 h. Then, the co-cultured cells were harvested by trypsynization and sorted using FACS Aria II (Becton Dickinson, USA) into two tubes: unstained macrophages and stained cancer cells (Figures 1D, E, F and 2).

### RNA isolation, validation, amplification, reverse transcription, labeling and hybridization

The sorted macrophages and cancer cells grown as the co-culture were centrifuged (2500 rpm for 5 min) in separated tubes, whereas cancer cells and macrophages grown as mono-cultures were washed with PBS and next scraped and centrifuged (2500 rpm for 5 min) in separated tubes. The total RNA from the samples was isolated using a Total RNA kit (A&A Biotechnology, Poland) according to the manufacturer's protocol. Isolated RNA samples were dissolved in RNase-free water. The quantity of RNA was measured using NanoDrop (NanoDrop Technologies, USA). The samples with adequate amounts of RNA were treated with DNaseI to eliminate a possibility of DNA contamination. The samples were subsequently purified using RNeasy MiniElute Cleanup Kit (Qiagen, Germany). Finally RNA samples were analyzed using BioAnalyzer (Agilent, USA) to measure the final RNA quality and integrity.

The Quick Amp Labeling Kit (Agilent, USA) was used to amplify and label target RNA to generate complementary RNA (cRNA) for oligo microarrays used in gene expression profiling and other downstream analyses. The gene expression of each cancer cell line, grown under co-culture conditions with macrophages, was compared against the gene expression of the same cancer cell line grown as a mono-culture (gene expression in CMT-U27 cell line grown as a co-culture with macrophages was compared to gene expression in CMT-U27 cell line grown as a mono-culture; gene expression in CMT-U309 cell line grown as a co-culture with macrophages was compared to gene expression in CMT-U309 cell line grown as a mono-culture; gene expression in P114 cell line grown as a co-culture with macrophages was compared to gene expression in P114 cell line grown as a mono-culture; gene expression in CMT-W1 cell line grown as a co-culture with macrophages was compared to gene expression in CMT-W1 cell line grown as a mono-culture; gene expression in CMT-W2 cell line grown as a co-culture with macrophages was compared to gene expression in CMT-W2 cell line grown as a mono-culture). The gene expression of macrophages grown as a co-culture with cancer cell lines was compared against the gene expression of macrophages grown as the mono-culture. Each sample was examined in a dye-swap to eliminate the effect of label factor. Thus, each biological condition was labelled once by Cy3 and once by Cy5. Taking the average of two labelled arrays, the dye effect on any particular gene was cancelled. The hybridization was performed with canine-specific AMADID Release GE 4 × 44 K microarrays (Agilent, USA) using Gene Expression Hybridization Kit (Agilent, USA) according to the manufacturer's protocol.

### Signal detection, quantification and analysis

Acquisition and analysis of hybridization intensities were performed using DNA microarray scanner (Agilent, USA). Then, the results were extracted using Agilent's Feature Extraction Software with normalization and robust statistical analyses. Results were analyzed for statistical purposes using Feature Extraction and Gene Spring software (Agilent, USA). The unpaired *t*-test with Benjamin-Hochberg FDR < 5% (false discovery rate) correction was applied (with p value cut-off < 0.01). For further analysis we hierarchically clustered the genes and chose only those with values within upper and lower cut-off (100.00 and 20.00, respectively) in each of the slide. We analyzed only genes that were regulated in all the examined samples within the group (that is: in all the cell lines grown with macrophages and in all the macrophages samples grown with cell lines) whose expression changed at least 3-fold in each of examined slide. In this experimental model we examined each of the sample in duplicate (dye-swap), whereas significant genes were chosen from five biological repetitions (five various cell lines). The area of the analyses covered in this publication has been deposited in NCBI's Gene Expression Omnibus and is accessible via GEO Series accession number GSE29339.

Gene function was identified using the PANTHER pathway analysis software [12] and Pathway Studio software (Agilent, USA). PANTHER on-line platform allowed for wide analysis of the *Canis familiaris *regulated genes and also for statistical analysis of number of regulated genes involved in specific pathways or biological functions compared to the normal healthy cell of this specie.

### Real-time qPCR

The mRNA sequences of the key genes were obtained from NCBI database. Primers were designed using PRIMER3 software (free on-line access) and checked using Oligo Calculator (free on-line access) and Primer-Blast (NCBI database). Primers' sequences are listed in Table 1. *HPRT *and *RPS19 *genes were used as non-regulated, reference genes for normalization of target gene expression [13,14]. Quantitative RT-PCR was performed using fluorogenic Lightcycler Fast Strand DNA Sybr Green (Roche) and the Light Cycler (Roche). The results were analyzed using comparative Ct method [15]. Relative transcript abundance of the gene equals ΔCt values (ΔCt = Ct^reference ^-Ct^target^). Relative changes in transcript are expressed as ΔΔCt values (ΔΔCt = ΔCt^control conditions^-ΔCt^co-culture conditions^). The experiment was conducted in triplicates.

**Table 1:** Primers used for real-time qPCR

Gene symbol	Forward primer	Reverse primer	Optimum annealing temp. (°C)	Optimum annealing time (sec)
*CCL2*	CTCCAGTCACCTGCTGCTAT	CACAGCTTCTTTGGGACACT	60	4

*CCL3*	CCAGGTCTTCTCACCATTTG	AGATAATACCGGGCTTGGAG	60	5

*CD163*	ATGTCCAGTGTCCAAAAGGA	CATGTGATCCAGGTCTCCTC	61	6

*CSF1R*	TGCAGTTTGGGAAGACTCTC	TGTGGACTTCAGCATCTTCA	60	4

*HIF1*	GATTGCAGCTCCATCTCCTA	TCCTTTTCCTGCTCTGTTTG	58	5

*IL18*	GATATGCCCGATTCTGACTG	GCCTGGAACACTTCTCTGAA	60	9

*MMP9*	CGACTACGACCAGGACAAAC	AAGCCCCACTTCTTGTCTCT	61	8

*VEGF-C*	CAGCAACACTACCACAGTGC	CTCCAGAATTTGAGGCAAAA	61	5

*Wnt7b*	GCGGAGGGCTGTGTATAAGA	GTCCCCTACTTTGCGGAACT	59	5

*HPRT*	AGCTTGCTGGTGAAAAGGAC	TTATAGTCAAGGGCATATCC	59	6

*RPS19*	CCTTCCTCAAAAAGTCTGGG	GTTCTCATCGTAGGGAGCAAG	61	10

Primers sequences used in this study and their annealing optimal temperature and time. The mRNA sequences of key genes were obtained from NCBI database. Primers were designed using PRIMER3 software (free on-line access) and checked using Oligo Calculator (free on-line access) and Primer-Blast (NCBI database). Primers sequences are listed in Table 1. *HPRT *and *RPS19 *genes were used as non-regulated reference genes for normalization of target gene expression [3,9]

### Immunohistochemistry (IHC)

The cells were cultured on Lab-Tek (Nunc Inc., USA) 4-chamber culture slides and were fixed with ethanol after 24 hrs.

The samples were incubated in the Peroxidase Blocking Reagent (Dako, Denmark) for 10 min at room temperature prior to the antibody incubation. After 30 min incubation in 5% bovine serum albumin (Sigma Aldrich, Germany), the rabbit polyclonal MCSF Receptor (other designations: MCSF-R or CSF-1R) obtained from Abcam (United Kingdom) primary antibodies were used (diluted in 1% bovine serum). According to the manufacturer's instructions the slides were incubated with antibodies at +4°C overnight. For the staining the EnVision kit (Labelled Polymers consist of secondary anti-rabbit antibodies conjugated with the HRP enzyme complex obtained from Dako) was used. To develop the coloured product, the 3,3'-Diaminobenzidine (DAB) substrate was used (Dako, Denmark). Finally, the haematoxylin was used for nuclei counterstaining.

Each slide was photographed 10 times using Olympus microscopy BX60. The colorimetric intensity of the CSF-1R expression reflected as IHC-stained antigen spots (brown colour) were counted by a computer-assisted image analyzer (Olympus Microimage™ Image Analysis, software version 4.0 for Windows, USA). The antigen spot colour intensity is expressed as a mean pixel optical density on a 1-256 scale.

### Confocal microscopy

The co-culture of cancer cells with macrophages was conducted as described above. The cancer cells grown as mono-culture and as a co-cultures were stained using Orange CellTracker fluorescent dye CMTMR, as described above. Cancer cells and macrophages grown as mono-cultures, as well as the co-culture were fixed with 70% ethanol (10 min) and washed three times in PBS. Cells were permeabilized with 0.5% Triton X-100/PBS (10 min), washed with PBS twice and incubated for 1 hr at room temperature with: mouse monoclonal anti-CD64 FITC-conjugated (Becton Dickinson, USA) antibodies (20 ul per 10^6 ^cells) and mouse monoclonal anti-CD14 FITC-conjugated (LifeSpan Biosciences, USA) antibodies (10 ul per 10^6 ^cells) according to the manufacturer's instructions. The cells were then washed three times with PBS and the coverslips were mounted on microscope slides using ICN mounting medium.

The cell imaging was performed by confocal laser scanning microscope FV-500 system (Olympus Optical Co, Germany). The combination of excitation/emission were: Argon 488 nm laser with 505-525 nm filter for FITC and HeNe 543 nm laser with 610 nm filter for CMTMR staining. The pictures were gathered separately for each fluorescence channel. The cells were examined using the Fluoview program (Olympus Optical Co., Germany).

### Wound-healing assay

To assess the ability to migration of cancer cells grown as a co-culture with macrophages, we applied a wound-healing test. The cancer cells (grown as the co-culture with macrophages and normal control cells) were separately seeded in multi-well plates and then (after 24 hrs when the cells were confluent), using a pipette tip (100 ul) a straight scratch has been made, simulating a wound. The images were captured at the beginning and at regular intervals (after 2, 4 and 6 hrs) during cell migration to close the wound. The images then were compared to quantify migration rate of the cells. This method is particularly suitable for studies of cell-cell interaction on cell migration [16]. The pictures has been analyzed using a computer-assisted image analyzer (Olympus Microimage™ Image Analysis, software version 4.0 for Windows, USA).

### Invasion assay

BD BioCoat Matrigel™ invasion chambers (BD Biosciences, USA) pre-coated with BD Matrigel matrix were used according to the manufacturer's protocol. The assay insert plates were prepared by rehydrating the BD Matrigel Matrix coating with phosphate buffered saline for two hrs at 37°C. The rehydration solution was carefully removed, 2.5 × 10^5 ^of control cancer cells or cancer cells grown as co-culture with macrophages (at the ratio of 10:1) was added to each apical chamber and 0.75 ml RPMI-1640 containing chemoattractant (10% FBS) was added to the basal chamber. Uncoated insert plates, included as invasion controls, were used without rehydration. Assay plates were incubated for 22 hrs at standard culturing conditions. 2.5 μg/ml Calcein AM were added to 20 μl DMSO and then, 10 μl was transferred to 12 ml Hanks Buffered Saline Dispense. 0.5 ml Calcein solution was then transferred into each well of 24-well plate. The medium from insert was removed and multiwell inserts were transferred to the plate containing 0.5 ml/well calcein. Plates were incubated an hour at standard culture conditions. The fluorescence of invaded cells was measured with excitation wave length 485 nm and emission wave length Em 530 nm using Tecan Infinite 200 Reader (Tecan, Switzerland).

### 3D culture

Cancer cells were treated with trypsin and resuspended in culture medium. 35 mm culture plates (Corning Inc., USA) were coated with 100 μl of growth factor reduced Matrigel (BD Biosciences, USA) and left to solidify for 30 min. at 37°C. The cells were then plated at a concentration of 10^4 ^cells/ml. The growth of cells on Matrigel was observed every day under phase-contrast microscope.

### Statistical analysis

The analysis for statistical purposes was conducted using Prism version 5.00 software (GraphPad Software, USA). The two-way ANOVA, ANOVA + Tukey HSD (Honestly Significant Difference) post-hoc test and *t*-test were applied. The p-value < 0.05 was regarded as significant whereas p-value < 0.01 and p-value < 0.001 as highly significant.

## Results

### Sorting of the co-cultured cells

Flow cytometry had easily distinguished the CMTMR-stained cells from the unstained macrophages (Figure 1D, E) and allowed a further proper sorting of each population (Figure 1F). The co-culture was maintained for at least 72 h. The differential staining prolonged for such period of time (Figure 1E, F and see also confocal microscopy results) showed no detrimental effect on proliferation and plating efficiency. The fluorescence intensity of the stained cancer cells after the 3-days co-culture with macrophages was the same as that of the control cancer cells grown as a mono-culture (Figure 1E, F; see also confocal microscopy results). Similar culture conditions had previously been described [17].

Our FACS sorting isolated a 97-99% pure population on postsort, with a positive result comparable to other reported data available [5]. Sorting purity was also assessed using fluorescence microscopy (showing no stained cells in macrophages tube and no unstained cells in cancers tube).

### Monocyte differentiation

**Figure 3:**
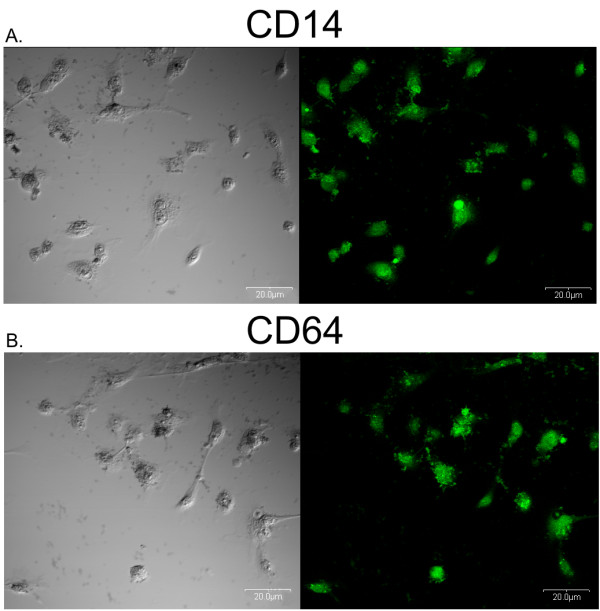
**Monocytes differentiation toward macrophages after 72 hrs of culture**. The pictures of CD64-positive monocytes sorted from mononuclear blood cells and cultured for 72 hrs showing their partial differentiation toward macrophages. (**A**) Macrophages grown as a mono-culture were stained using anti-CD64 FITC-conjugated antibodies (Becton Dickinson, USA). (**B**) Macrophages grown as a mono-culture were stained using anti-CD14 FITC-conjugated antibodies (LifeSpan Biosciences, USA). Cells were pictured by confocal laser scanning microscope FV-500 system (Olympus Optical Co, Germany). The cells were examined using the Fluoview program (Olympus Optical Co., Germany).

The morphological assessment of the monocytes culture (obtained from the sorted CD64-positive monocytes) using confocal microscopy confirmed that after the 72 hrs they were partially differentiated into macrophages (Figure 3) despite no stimulation with growth factors. These results are consistent with the data available on the subject, showing that the 3-day monocyte culturing leads to their partial differentiation toward the macrophages phenotype [10,11]. The colonies were stained using anti-CD14, and anti-CD64 antibodies (Figure 3). Analysis of stained cells and image contrast obtained with Nomarski Interference Contrast (representative pictures are shown at Figure 3) showed that all the macrophages expressed CD14 antigen as well as CD64 what can also confirm monocyte differentiation towards macrophages [18].

### Global gene expression analysis

**Figure 4:**
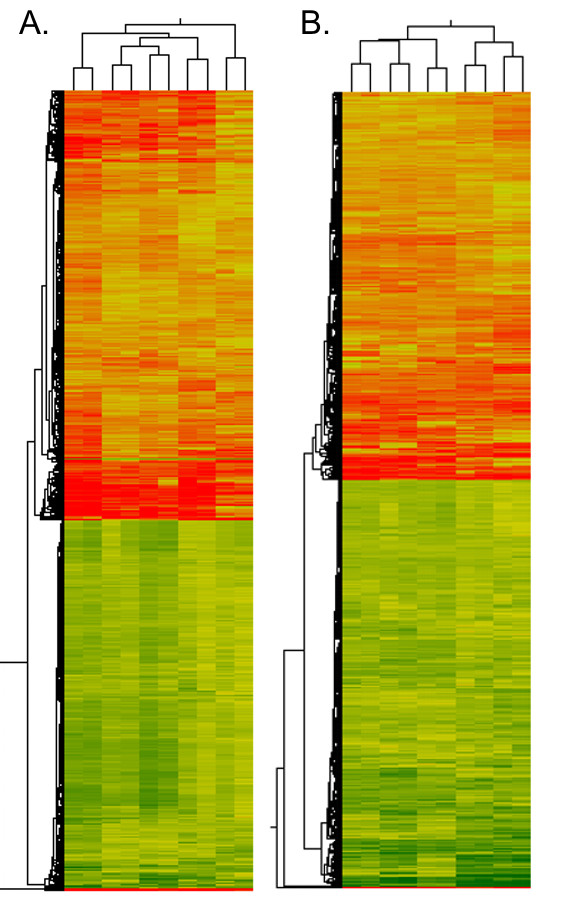
**Hierarchical clustering of gene expression in canine mammary cancer cell lines and macrophages grown as a co-culture**. Gene Spring (Agilent, USA) diagrams of gene expression clustering of (**A**) canine mammary cancer cell lines grown as a co-culture with macrophages and (**B**) macrophages grown as a co-culture with cancer cells in both microarray experiments (dye-swaps) shows highly repeatable results. Each row represents a single gene, and each column an experimental sample (from the left: at A. panel: CMT-U27, CMT-U309, P114, CMT-W1 and CMT-W2 cell lines grown with macrophages; at B. panel: macrophages grown with CMT-U27, CMT-U309, P114, CMT-W1 and CMT-W2 cell lines). In each sample, the ratio of the abundance of transcripts of each gene to the mean abundance of the gene's transcript, among all the samples is represented by the colour: green squares, transcript levels below the mean; red squares, transcript levels greater than the mean. Colour saturation reflects the magnitude of the ratio relative to the mean for each set of samples. Dendrogram represents similarities in the expression patterns between experimental samples.

The Gene Spring (Agilent, USA) hierarchical clustering depicted similar gene expression in each of the dye-swap experiments (Figure 4) what indicates that all microarray samples were successfully labelled, hybridized, scanned and that they are highly reproducible. The unpaired *t*-test with Benjamin-Hochberg FDR < 5% (false discovery rate) correction (with p value cut-off < 0.01) and further PANTHER analysis revealed 43 up-regulated and 4 down-regulated genes in cancer cells grown in co-culture with macrophages with values within upper and lower cut-off (100.00 and 20.00, respectively) in each of the slide (Table 2). Only the genes whose expression had changed at least 3-fold in each of the examined slide were chosen for further analyses. These up/down-regulated genes were common for each cell line examined individually, when compared with the same cell line grown as a mono-culture.

**Table 2:** Up/down-regulated genes in canine mammary cancer cell lines grown as co-culture with macrophages

**No**.	Fold change	Gene symbol	Gene name
1	↑3.25	ANPEP	Aminopeptidase N

2	↑3.83	ATP6V0E1	V-type proton ATPase subunit e 1

3	↑4.98	C5AR1	C5a anaphylatoxin chemotactic receptor

4	↑4.86	CCL2	C-C motif chemokine 2

5	↑5.23	CCL3	C-C motif chemokine 3

6	↑4.28	CCL4	C-C motif chemokine 4

7	↑3.99	CCL5	C-C motif chemokine 5

8	↑3.91	CCL8	C-C motif chemokine 8

9	↑3.03	CCR1	CCR1

10	↑3.66	CCR5	C-C chemokine receptor type 5

11	↑4.47	CD163	Soluble CD163

12	↑3.55	CD40	Tumor necrosis factor receptor superfamily member 5

13	↑4.89	CD80	CD80

14	↑4.31	CD86	CD86

15	↑3.38	COX17	Cytochrome c oxidase copper chaperone

16	↑3.62	CSF2	Granulocyte-macrophage colony-stimulating factor

17	↑3.53	CSF3	Granulocyte colony-stimulating factor

18	↑3.68	CSF1R	Colony stimulating factor receptor 1

19	↑3.09	CTSC	Dipeptidyl-peptidase 1 light chain

20	↑5.06	CTSK	Cathepsin K

21	↑5.92	CTSS	Cathepsin S

22	↑3.5	CXCR7	C-X-C chemokine receptor type 7

23	↑3.26	DLA-64	DLA-64

24	↑3.26	EMR1	EMR1

25	↑3.31	FTH1	Ferritin heavy chain

26	↑3.1	FTL	Ferritin light chain

27	↑5.25	IL1A	Interleukin-1 alpha

28	↑5.07	IL1B	Interleukin-1 beta

29	↑3.45	IL6	Interleukin-6

30	↑5.6	IL8	Interleukin-8

31	↑3.09	MMP9	Matrix metalloproteinase-9

32	↑4.83	MX1	Interferon-induced GTP-binding protein Mx1

33	↑3.34	NPC2	Epididymal secretory protein E1

34	↑3.19	OOEP	Oocyte-expressed protein

35	↑3.6	PLA2G7	Platelet-activating factor acetylhydrolase

36	↑3.23	PSAP	Pulmonary surfactant-associated protein A

37	↑4.84	PSMB8	Proteasome subunit beta type-8

38	↑3.57	PSMB9	Proteasome subunit beta type

39	↑3.74	PTGES	Prostaglandin E synthase

40	↑3.35	SLAMF1	Signaling lymphocytic activation molecule

41	↑3.34	SYK	SYK

42	↑4.64	TLR2	Toll-like receptor 2

43	↑3.55	TLR4	TLR4

44	↓3.11	RXRB	Retinoic acid receptor RXR-beta

45	↓3.17	SLC5A3	Sodium/myo-inositol cotransporter

46	↓3.06	MSX2	Homeobox protein MSX-2

47	↓5.17	KRT13	Keratin 13

The list of up- (↑) and down- (↓) regulated genes in canine mammary cancer cell lines grown as co-culture with macrophages. The unpaired *t*-test with Benjamin-Hochberg FDR < 5% (false discovery rate) correction (with p value cut-off < 0.01) (Gene Spring, Agilent, USA) and further PANTHER analysis were conducted

The unpaired *t*-test with Benjamin-Hochberg FDR < 5% (false discovery rate) correction (with p value cut-off < 0.01) and further PANTHER analysis revealed 30 up-regulated and 25 down-regulated genes in monocytes/macrophages grown as co-culture with cancer cells with values within upper and lower cut-off (100.00 and 20.00, respectively) in each of the slide (Table 3). Only the genes whose expression had changed at least 3-fold in each of examined slide were chosen for further analyses. These differentially expressed genes were common for macrophages grown with each cell line individually in comparison to macrophages grown as the mono-culture.

**Table 3:** Up/down-regulated genes in macrophages grown as a co-culture with canine mammary cancer cells

No	Fold change	Gene symbol	Gene name
1	↑5.78	ABCC5	ABCC5

2	↑3.17	ANXA2	Annexin A2

3	↑3.22	ASPM	Abnormal spindle-like microcephaly-associated protein homolog

4	↑3.0	BGN	Biglycan

5	↑5.83	CAV1	Caveolin-1

6	↑4.04	CAV2	Caveolin-2

7	↑3.47	CCL20	CCL20

8	↑3.15	COL1A1	Collagen alpha-1(I) chain

9	↑4.51	CSF2	Granulocyte-macrophage colony-stimulating factor

10	↑4.28	CXCL10	C-X-C motif chemokine 10

11	↑4.06	EGFR	EGFR

12	↑4.66	FZD6	Frizzled-6

13	↑4.29	GATA6	GATA6

14	↑3.79	IL12A	Interleukin-12 subunit alpha

15	↑3.63	IL13RA2	Interleukin-13 receptor alpha-2 chain

16	↑5.39	MMP3	Stromelysin-1

17	↑4.58	MYC	Myc proto-oncogene protein

18	↑3.51	OAT	OAT

19	↑6.54	PFKM	6-phosphofructokinase, muscle type

20	↑3.86	PTPLA	Protein-tyrosine phosphatase-like member A

21	↑3.36	RAD51	DNA repair protein RAD51 homolog 1

22	↑4.86	RHPN2	Rhophilin-2

23	↑3.05	RPL23	60S ribosomal protein L23

24	↑3.1	RPS17	40S ribosomal protein S17

25	↑3.54	RPS18	40S ribosomal protein S18

26	↑4.87	UACA	Uveal autoantigen with coiled-coil domains and ankyrin repeats

27	↑5.11	Wnt5b	wingless-type MMTV integration site family member 5b

28	↑3.78	Wnt7a	wingless-type MMTV integration site family member 7a

29	↑4.51	Wnt7b	wingless-type MMTV integration site family member 7b

30	↑4.11	YES1	Proto-oncogene tyrosine-protein kinase Yes

31	↓3.63	ANPEP	Aminopeptidase N

32	↓3.27	APOE	Apolipoprotein E

33	↓5.73	C5AR1	C5a anaphylatoxin chemotactic receptor

34	↓5.24	CCL13	C-C motif chemokine 13

35	↓3.33	CCL2	C-C motif chemokine 2

36	↓3.36	CCR1	CCR1

37	↓3.73	CCR5	C-C chemokine receptor type 5

38	↓3.66	CD86	CD86

39	↓3.1	CTSK	Cathepsin K

40	↓3.08	CXCR7	C-X-C chemokine receptor type 7

41	↓3.84	DHDH	dehydrogenase

42	↓4.1	EDNRB	Endothelin B receptor

43	↓4.14	HTR7	HTR7

44	↓3.47	IL18	Interleukin-18

45	↓3.15	IL1B	Interleukin-1 beta

46	↓3.1	KCNJ2	Inward rectifier potassium channel 2

47	↓3.61	MMP9	Matrix metalloproteinase-9

48	↓4.72	MYH7	Myosin-7

49	↓4.19	OR04A01	olfactory receptor

50	↓3.64	PDPN	Podoplanin

51	↓3.04	SLC11A1	Natural resistance-associated macrophage protein 1

52	↓3.08	SLC46A2	Thymic stromal cotransporter homolog

53	↓4.09	SLC8A1	Sodium/calcium exchanger 1

54	↓3.15	TLR2	Toll-like receptor 2

55	↓3.11	TLR4	Toll-like receptor 4

The list of up- (↑) and down- (↓) regulated genes in macrophages grown as co-culture with canine mammary cancer cells. The unpaired *t*-test with Benjamin-Hochberg FDR < 5% (false discovery rate) correction (with p value cut-off < 0.01) (Gene Spring, Agilent, USA) and further PANTHER analysis were conducted

### Over- representation of pathways in cells grown under co-culture conditions

The PANTHER binomial statistics tool allowed us to statistically determine over-manifestation of PANTHER pathways classification categories.

The 15 significantly over-manifested (p < 0.05) pathways were observed in cancer cells grown as a co-culture (Table 4). Most of the up-regulated genes in cancer cells lines were involved in the stimulation of 1. inflamation mediated by chemokine and cytokine signaling pathways, 2. Toll receptor signalling pathway (7 genes, p = 9.44E-06), and 3. B cell activation (7 genes, p = 6.8E-05).

**Table 4:** Genes involved in over-manfested cellular pathways in canine mammary cancer cells grown as a co-culture with macrophages

Pathway	number of genes	P-value
Inflammation mediated by chemokine and cytokine signaling pathway	21	1.81E-11

Toll receptor signaling pathway	7	9.44E-06

B cell activation	7	6.80E-05

Purine metabolism	3	1.04E-04

T cell activation	8	1.60E-04

Acetate utilization	2	6.87E-04

Apoptosis signaling pathway	6	3.86E-03

Adenine and hypoxanthine salvage pathway	2	8.65E-03

Interleukin signaling pathway	6	8.68E-03

Plasminogen activating cascade	2	1.19E-02

5-Hydroxytryptamine degredation	2	2.19E-02

Lysine biosynthesis	1	2.48E-02

Salvage pyrimidine deoxyribonucleotides	1	3.69E-02

PDGF signaling pathway	5	3.99E-02

Methylcitrate cycle	1	4.89E-02

The list of over-manifested cellular PANTHER pathways in cancer cells grown as co-cultures with macrophages. The binomial test for each PANTHER pathway was used

Other important over-manifested pathways are apoptosis signaling pathway (6 genes, p = 3.86E-04), interleukin signaling pathway (6 genes, p = 8.86E-03) and PDGF signaling pathway (5 genes, p = 3.99E-02). The down-regulated genes in the cancer cells were not involved in any significantly over- manifested pathways.

The up-regulated genes in the macrophages were involved in 18 over-manifested pathways (Table 5): angiogenesis (13 genes, p = 1.17E-04), p53 pathway feedback loops2 (6 genes, p = 2.16E-04), Wnt signaling pathway (17 genes, p = 2.90E-04). Other important pathways are: inflammation mediated by chemokine and cytokine signaling pathway (12 genes, p = 0.09E-03) and FGF signaling pathway (6 genes, p = 4.05E-02).

**Table 5:** Genes involved in over-manifested cellular pathways in macrophages grown as a co-culture with canine mammary cancer cells

Pathway	number of genes	P-value
Angiogenesis	13	1.17E-04

p53 pathway feedback loops 2	6	2.16E-04

Wnt signaling pathway	17	2.90E-04

Alzheimer disease-presenilin pathway	9	1.04E-03

Cadherin signaling pathway	9	1.55E-03

p53 pathway	8	1.63E-03

Plasminogen activating cascade	3	2.84E-03

TGF-beta signaling pathway	8	5.07E-03

Inflammation mediated by chemokine and cytokine signaling pathway	12	9.09E-03

Arginine biosynthesis	2	1.29E-02

Alzheimer disease-amyloid secretase pathway	5	1.77E-02

Alpha adrenergic receptor signaling pathway	3	2.68E-02

Metabotropic glutamate receptor group I pathway	3	3.95E-02

FGF signaling pathway	6	4.05E-02

Succinate to proprionate conversion	1	4.16E-02

PLP biosynthesis	1	4.16E-02

Endothelin signaling pathway	5	4.29E-02

Muscarinic acetylcholine receptor 1 and 3 signaling pathway	4	4.67E-02

The list of over-manifested cellular PANTHER pathways in macrophages grown as co-cultures with canine mammary cancer cells. The binomial test for each PANTHER pathway was used

### Over-representation of genes involved in particular biological processes in cells grown under co-culture conditions

The PANTHER binomial statistics tool allowed us to statistically determine over-manifestation of PANTHER biological processes classification categories. The most important biological processes in cancer cells grown as a co-culture were: macrophage activation (21 genes, p = 6.24E-11), cell motion (28 genes, p = 7.49E-06), mammary gland development (5 genes, p = 2.17E-03), cell-cell adhesion (19 genes, p = 5.06E-03), angiogenesis (11 genes, p = 9.51E-03).

The most important biological processes in macrophages grown as a co-culture were cell-matrix adhesion (10 genes, p = 3.92E-03) and cell-cell adhesion (28 genes, p = 5.79E-03).

### The results were confirmed at mRNA level using real-time qPCR analysis

For the purposes of the microarray data validation, we have selected 9 genes that may play the most important role in cancer cells-macrophages interactions: CCL2, CCL3, CD163, CSF1R, HIF1, Il-18, MMP9, VEGF-C, and Wnt7b. Real-time qPCR results showed similar trends in gene expression modulations as were observed in microarray studies. The real-time qPCR confirmed the CCL2, CCL3, CD163, CSF1R, MMP9, HIF1, VEGF-C up-regulation in cancer cells grown as a co-culture with macrophages. The analysis also confirmed down-regulation of CCL2, IL18, and up-regulation of Wnt7b genes in macrophages grown under co-culture conditions with cancer cells (Table 6).

**Table 6:** Fold change of genes randomly selected for confirmation of microarray results

	*Wnt7b*	*IL18*	*CCL2*	*CCL3*	*CD163*	*CSF1R*	*HIF1*	*MMP9*	*VEGF-C*
*MQ co-culture with CMT-U27 v.s. monoculture*	↑2.08	↓111.1	↓58.8						

*MQ co-culture with CMT-U309 v.s. monoculture*	↑2.27	↓1.5	↓9.1						

*MQ co-culture with P114 v.s. monoculture*	↑1.45	↓6.3	↓8.3						

*MQ co-culture with CMT-W1 v.s. monoculture*	↑6.67	↓142.9	↓22.7						

*MQ co-culture with CMT-W2 v.s. monoculture*	↑1.61	↓62.5	↓13.3						

*CMT-U27 co-culture with MQ v.s. monoculture*			↑407.3	↑1.93	↑58.08	↑229.9	↑9.1	↑711.6	↑28.1

*CMT-U309 co-culture with MQ v.s. monoculture*			↑1612.4	↑1.01	↑48.67	↑129.8	↑1.4	↑207.9	↑1.2

*P114 co-culture with MQ v.s. monoculture*			↑931.0	↑1269.46	↑17.99	↑64.1	↑1.7	↑71.3	↑1.9

*CMT-W1 co-culture with MQ v.s. monoculture*			↑333.8	↑101.13	↑11.87	↑9.3	↑3.0	↑209.8	↑7.7

*CMT-W2 co-culture with MQ v.s. monoculture*			↑109.9	↑51.09	↑31.25	↑8.3	↑5.0	↑147.3	↑30.4

Fold change of genes randomly selected for confirmation of microarray results in macrophages grown as a co-culture with canine mammary cancer cell lines (versus macrophages grown as a monoculture) and in canine mammary cancer cell lines grown as a co-culture with macrophages (versus canine mammary cancer cell lines grown as a monoculture)

### Confocal microscopy and IHC analysis revealed myeloid-lineages markers expression in cancer cells following the co-culturing with macrophages

**Figure 5:**
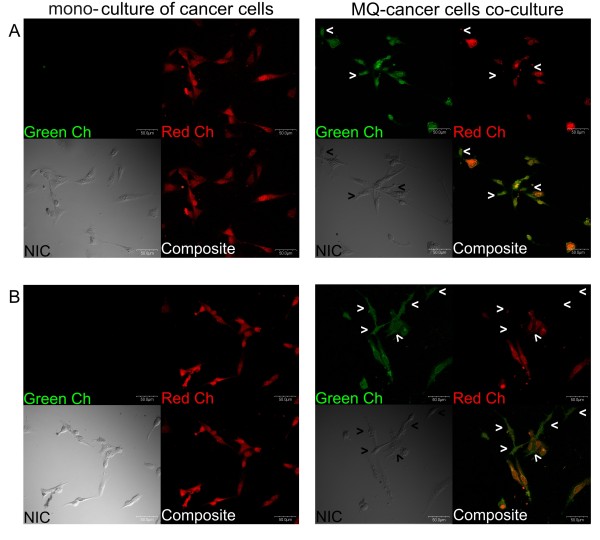
**Macrophage-specific antigens expression in cancer cells in the co-culture conditions**. Representative pictures obtained using confocal microscopy showing CD64 (**A**) and CD14 (**B**) expression (green fluorescence) in CMT-W2 canine mammary cancer cell line (stained using CMTMR) grown as a mono-culture (left panel) and as a co-culture with macrophages (right panel) for 72 hrs. Macrophages are marked by arrows. Cell imaging was performed by confocal laser scanning microscope FV-500 system (Olympus Optical Co, Germany). The cells were examined using the Fluoview program (Olympus Optical Co., Germany).

The microarray analysis revealed that the co-culturing of cancer cells with macrophages initiated the myeloid-specific antigens expression in cancer cells. Thus, to confirm this data the confocal analysis was conducted. The expression of two monocytes/macrophages-specific antigens was assessed: CD14 and CD64. The cancer cells grown as mono-culture did not show expression of both antigens (Figure 5A, B left panel), whereas the strong expression of these two markers was detected when the cancer cells were co-cultured with macrophages (Figure 5A, B right panel). The confocal observations also showed that macrophages co-exist closely with the cancer cells, even forming cell fusions.

**Figure 6:**
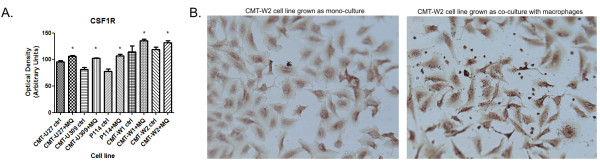
**CSF1R expression in canine mammary cancer cell lines in control conditions and after the co-culture with macrophages**. (**A**) The graph of mean optical density of CSF1R (and SD) in canine mammary cell lines (CMT-U27, CMT-U309, P114, CMT-W1 and CMT-W2) grown in control conditions (ctrl) and as a co-culture with macrophages (+MQ). (**B**) Representative pictures of CSF1R expression in CMT-W2 cell line (in control conditions and after co-culture with macrophages) obtained using Olympus BX60 microscope (at the magnification of 200×). The CSF1R is reflected as brown colour. Ten pictures in each slide were analyzed. The colorimetric intensity of the IHC-stained antigen spots was counted by a computer-assisted image analyzer (Olympus Microimage™ Image Analysis, software version 4.0 for Windows, USA) and the antigen spot colour intensity is expressed as mean pixel optical density on a 1-256 scale. The statistical analysis was performed using Prism version 5.00 software (GraphPad Software, USA). The unpaired *t*-test was applied to analyze the optical density in cell lines. p < 0.05 was regarded as significant and marked as *.

IHC analysis also confirmed acquisition of macrophage's marker by cancer cells after co-culture. The expression of CSF-1R antigen was significantly higher (p < 0.05) in all the cancer cell lines after co-culturing with macrophages (Figure 6, Table 7). Thus, the co-culture conditions induced or increased macrophage antigens expression by cancer cells.

**Table 7:** CSF1R expression at protein level in canine mammary cancer cell lines

Cell line	CMT-U27 ctrl	CMT-U27 +MQ	CMT-U309 ctrl	CMT-U309 +MQ	P114 ctrl	P114 +MQ	CMT-W1 ctrl	CMT-W1 +MQ	CMT-W2 ctrl	CMT-W2 +MQ
Mean Optical Density (Arbitrary Units)	95.40	106.60	81.28	102.30	77.99	107.20	114.50	135.40	119.10	132.00

SD	4.99	2.23	5.59	1.35	5.82	4.12	15.77	4.86	6.34	4.87

The CSF1R expression in canine mammary cancer cell lines grown as mono-culture (ctrl) and as co-culture with macrophages (+MQ). Results are presented as mean optical density ± SD

### Migration assay

**Figure 7:**
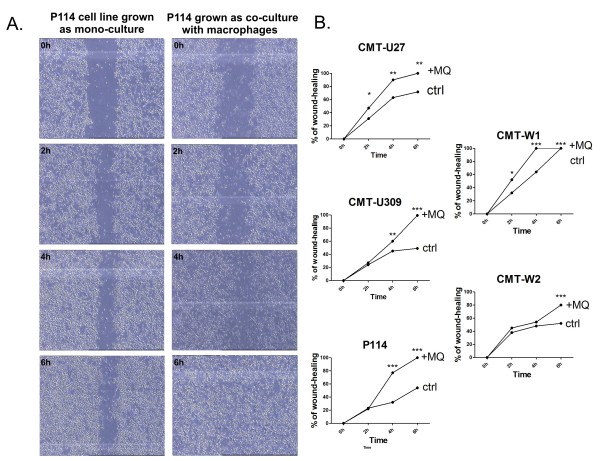
**Wound healing assay of canine mammary cancer cells grown in control conditions and as a co-culture with macrophages**. (**A**) Representative pictures of migration (wound closing) of P114 cell line grown as a mono-culture and co-culture with macrophages at 0, 2, 4 and 6 hrs after the scratch was made. (**B**) The graphs of % of wound closure after the 2, 4 and 6 hrs of migration. The pictures were taken using phase-contrast microscopy (Olympus). The statistical analysis was performed using Prism version 5.00 software (GraphPad Software, USA). The one-way ANOVA was applied to analyze the results. p < 0.05 was regarded as significant and marked as *, whereas p < 0.01 and p < 0.001 were regarded as highly significant and marked as ** and ***, respectively.

The wound healing assay showed that in all of the cancer cell lines the co-culturing with macrophages increased their migratory abilities (Figure 7). CMT-U27 cells grown with macrophages completely closed the wound (100%) in 6 hrs, whereas CMT-U27 control cells after 6 hrs closed 71.5% of the wound. Similarly, CMT-U309 and P114 cells (grown with macrophages) after 6 hrs almost completely closed the wound (99.2% and 99.9%, respectively), whereas control cells closed only 49% and 54% (respectively) of the wound. CMT-W1 cells grown with macrophages completely closed the wound after 4 hrs, whereas CMT-W1 control cells after 6 hrs (after 4 hrs 64% of the wound was closed). CMT-W2 cells grown with macrophages closed 80% of the wound after 6 hrs, whereas control cells closed only 52% of the wound.

### Invasion assay and 3D culture

**Figure 8:**
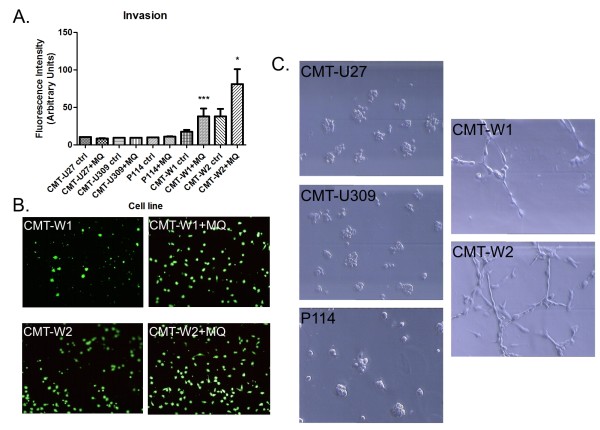
**Invasion of canine mammary cancer cell lines and growth characteristics in Matrigel matrix**. (**A**) The graph of fluorescence intensity related to invasion of canine mammary cancer cell lines (CMT-U27, CMT-U309, P114, CMT-W1 and CMT-W2) grown as a mono-culture (ctrl) and as a co-culture with macrophages (+MQ). The statistical analysis was performed using Prism version 5.00 software (GraphPad Software, USA). The unpaired *t*-test was applied to analyze the results. p < 0.05 was regarded as significant and marked as * whereas p < 0.001 was regarded as highly significant and marked as ***. (**B**) Pictures of highly invaded CMT-W1 and CMT-W2 cells (green fluorescence) obtained using Olympus BX60 microscope examined in control conditions and after co-culturing with macrophages. (**C**) Growth characteristics of CMT-U27, CMT-U309, P114, CMT-W1 and CMT-W2 cell lines (phase contrast micrographs) grown on Matrigel matrix for 24 hrs.

Invasion assay showed that not all of the cancer cells have migratory abilities. Only CMT-W1 and CMT-W2 control cells migrated throw the Matrigel (Figure 8A, B). The co-culturing of these cells for 72 hrs with macrophages significantly increased their migratory abilities (Figure 8A, B). The fluorescence intensity related to the CMT-W1 control cells migration was 17.57, whereas to the CMT-W1 cell line grown with macrophages was 38.08 (p < 0.001). The fluorescence intensity related to the CMT-W2 control cells migration was 38.00, whereas to the CMT-W2 cell line grown with macrophages was 80.75 (p < 0.05).

To confirm the ability of these cell lines to matrix invasion, we have assessed their growth characteristics on Matrigel matrix (Figure 8C). After 22 hrs of culturing (similarly as in the invasion assay) on Matrigel CMT-U27, CMT-U309 and P114 cell lines formed colonies, whereas CMT-W1 and CMT-W2 cell lines formed branching structures (Figure 8C) what indicated their invasive phenotype. The culture was maintained for 1 week, showing that after 5 days also P114 cell line formed slight branches.

## Discussion

It has been evident over the last few years that macrophages play an important role (via various factors) in tumour cell invasion of the normal surrounding tissues, cancer proliferation and metastasis to local and distant sites [19]. All the interactions within the tumour are intricately complex and a better understanding of them would require some further exploration of the underlying molecular processes.

**Figure 9:**
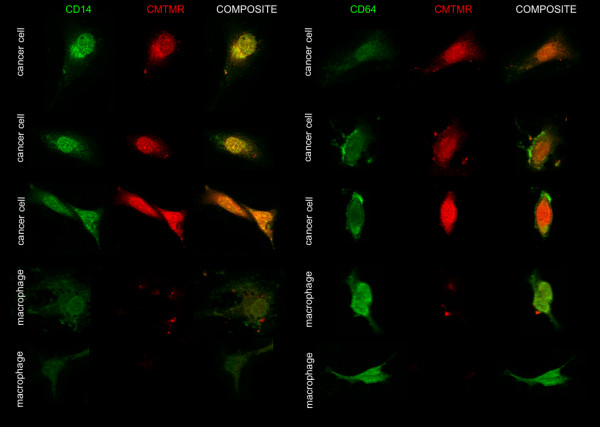
**Co-cultured cells staining characteristics**. Pictures of representative cells (canine mammary cancer cells and macrophages) obtained using confocal microscopy showing CD14 and CD64 expression (green fluorescence) and red-stained cytoplasms of cancer cells (stained using CMTMR) grown as a co-culture for 72 hrs. Cell imaging was performed by confocal laser scanning microscope FV-500 system (Olympus Optical Co, Germany). The cells were examined using the Fluoview program (Olympus Optical Co., Germany).

In this article, we demonstrated that co-culturing of canine mammary cancer cells and macrophages initiate a dynamic 'chemical conversation' between the tumour cells and macrophages. They also start to behave as one organism. Many of the previous studies have been conducted using cancer cells and macrophages grown without direct contact in trans-well inserts or modified Boyden chambers [10], however there are also very few reports on direct co-culturing of macrophages and cancer cells [20-22]. Despite that direct co-culturing of macrophages and cancer cells (with cell-cell contact) can be a good experimental model, because it reflects *in vivo *conditions more vividly (where all the cells have direct contact) than co-culturing where the cells can contact via soluble factors only, direct co-culturing brings about the risk of macrophages contamination by cancer cells owing to macrophages taking up fragments of cancer cells. To assess the possible risk of artificial sorting of macrophages (that digested red-stained cancer cells) as cancer cells (CMTMR-positive group) we checked our post-sort population using fluorescence microscopy (no red-stained cells in macrophages tube and no unstained cells in cancer cells tube have been seen, data not shown). Our co-culture has also been assessed using confocal microscopy (Figures 5 and 9). It showed that cancer cells were perfectly bright stained with CMTMR (whole cytoplasms was regularly red-stained). These cells also showed green-stained pattern due to the macrophages-specific antigen expression. Thus, we confirmed that we sorted the population of cancer cells showing macrophages-specific antigens. We have also observed very few macrophages with very small amount of red dye in their phagosomes which probably were digested fragments of cancer cells (Figure 9), but we observed attentively only the macrophages without any red dye. During cell sorting we only took cells with high PerCP-Area red-signal (Figure 1F) and gated for sorting only the high-positive cancer cells as opposed to macrophages with low signal. Even if some macrophages happen to have red dye in phagosome, they would have been somewhere between these two gates or qualified as macrophages. Bearing in mind that the possibility of contamination of macrophages RNA samples by cancer RNA is even less, as macrophages produce ribonucleases [23] that immediately degrade any digested (unstable) RNA molecules. Moreover, the possibility that macrophages can take up cancer cells during *in vitro *culturing is as likely as in a naturally occurring tumour *in vivo*, but researchers run a risk because the problem is extremely interesting and requires investigation [24,25].

The present microarray analysis of five various canine mammary cancer cell lines and canine macrophages grown together, revealed significant changes in genes expression in comparison to the same cells grown as mono-cultures.

Under co-culture conditions the cancer cells express the macrophages-specific antigens, e.g. CD14, CD64, CD163, CSF1R (Table 3, Figures 5, 6 and 9). According to subject literature, CD163 is expressed not only by normal monocytes/macrophages but also by neoplasms [26,27]. CD163-positive cancers had more severe histological aberrations due to genomic instability. Thus, it is hypothesized [26] that tumour cells express atypical genes (specific for macrophages) due to genomic instability. This may be caused by the presence of various proteins secreted by macrophages and changes induced by them on the microenvironment. Another theory explaining why cancer cells may exhibit myeloid cells-specific antigens is that the cells fuse, forming hybrids that adopt phenotypic features of both parental cells [28]. Cancer cells may fuse spontaneously with several types of somatic cells [29-32]. Some authors suggest that the result of cancer-myeloid cell fusion is the hybrid with a metastatic phenotype [33-39]. The tumour cells that express myeloid antigens may also exhibit other phenotypic characteristics of macrophages, such as capabilities of cell rolling, spreading, dissociation, diapedesis, migration and matrix invasion. The metastatic cancer cells have all these capacities because the process of metastasis requires a coordinated steps promoting angiogenesis, controlling adhesion, proteolysis and motility. Our confocal imaging and IHC examination has proven that under co-culture conditions expression of macrophages markers (CD14, CD64, CSF1R) in almost all of the cancer cells was initiated. Moreover, migration and invasion assays showed that the presence of macrophages in cancer microenvironment triggers migration in all of the cancer cell lines. The co-culturing of macrophages increased invasion in the cell lines that show high invasive abilities in control conditions. The ability of CD163 receptor (and perhaps other macrophage-specific antigens) to trigger the production of pro-inflammatory mediators [40,41] may be a key factor that brings changes to the cancer cell biology and stimulates it to cytokines/chemokines/growth factors production for further myeloid cell attraction.

Our microarray analysis of cancer cells grown with macrophages revealed the over-manifestation of genes activity which are involved in macrophages-cancer cells 'conversation'. We found up-regulation of the highly potent macrophage attracting factors: CCL2 (MCP-1), CCL3 (MIP-1α), CCL4 (MIP-1β) and CCL5 (RANTES) as well as CCR5 in cancer cells grown as co-culture with macrophages. Recent studies have demonstrated that CCL2 acts directly (via CCR5) in an autocrine manner on several human carcinomas regulating the migration and invasive properties of tumour cells [42,43]. Furthermore, CCL3-CCR5 axis can increase the MMP-9 expression contributing to angiogenesis, ECM degradation and metastasis. Our gene expression analysis seems to support both hypotheses, as besides CCL2, CCL3 and CCR5 up-regulation, we also observed increased expression of MMP-9 in cancer cells grown under co-culture conditions with macrophages.

We found down-regulation of the pro-inflammatory CD163 in macrophages grown as co-culture with cancer cells. We also found down-regulation of several key inflammation cytokines and macrophage activators, such as: CCL2, CCL13, CCR1, and CCR5; whereas up-regulation of other inflammation cytokines: CXCL10, CSF-2. Decrease of the expression of these genes in macrophages grown with cancer cells as co-cultures may be induced by hypoxia. Down-regulation of CCL2, CCR1 and CCR5 genes in macrophages under hypoxic conditions has been demonstrated by various authors [44-46]. This phenomenon has a biological explanation, as decrease of CCL2, CCR1 and CCR5 expression inhibits chemotaxis signalling what in turn prevents TAMs from leaving hypoxic areas [47] and triggers their angiogenic effect (e.g. by CXCL10 chemokine). Up-regulation of HIF-1 in cancer cells may indicate hypoxic conditions in co-culture (similarly as in tumour). Hypoxia additionally regulates angiogenesis by up-regulating VEGF-C in cancer cells and it initiates their epithelial-mesenchymal transition (EMT) [48].

**Figure 10:**
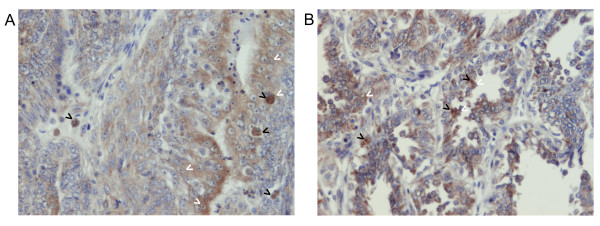
**The expression of CSF1R and CD14 in canine mammary cancer tissues**. Pictures obtained using Olympus BX60 microscope (x200) showing CSF1R (**A**) and CD14 (**B**) expression in canine mammary cancer tissues. These macrophage-specific antigens were detected not only on the monocytes/macrophages, but also on the cancer tissue (reflected as brown colour). Black arrows indicate macrophages, whereas white arrows indicate brown-coloured cancer cells. Tissue sections were stained using rabbit polyclonal CSF1R antibody and rabbit polyclonal CD14 antibody (both obtained from Abcam, United Kingdom). For the staining the EnVision kit (Dako, Denmark) was used (Labelled Polymers consist of secondary anti-rabbit antibodies conjugated with the Horseradish peroxidase HRP enzyme complex). To develop the coloured product, the 3,3'-Diaminobenzidine (DAB) substrate was used. Finally, the haematoxylin was used for nuclei counterstaining. The tissue details, procedure, and results have been described previously [20].

Our gene expression analysis also revealed up-regulation of CSF-2 (GM-CSF) in macrophages grown as co-culture with cancer cells and CSF-2, CSF-3 and CSF1R up-regulation in cancer cells grown together with macrophages. CSFs are potent factors regulating the survival and differentiation of macrophages being their chemoattractants [49]. The CSF-1R expression in normal mice and humans is limited to macrophages [49]. However, in many tumours CSF-1R and CSFs are also expressed in cancer cells. CSF-1R expression at protein level was also confirmed in this study (Figure 6). For example, co-expression of CSF-1 and its receptor can be found in 50% of late stage breast and 70% of endometrial cancers [50,51]. During the course of our previous studies we found the CSF-1R expression in canine mammary cancer cells (Figure 10), as well as in tumour-associated macrophages [52]. The results of the studies of Kirma et al. [53] suggest that the CSFs might have an autocrine role through CSF1R in epithelial tumour cells promoting their invasiveness into the surrounding matrix [54,55].

All genes given above are important to entrap macrophages in malignant tumours [56].

In macrophages grown as co-culture with cancer cells expression of three ligands of Wnt pathway increased significantly: Wnt5b, Wnt7a and Wnt7b. Although the Wnt activation has been described by many authors in various cancer cells [57,58], only one study, so far [5], described the Wnt activation in macrophages taken from mice tumours. The authors described mechanism, by which macrophages stimulate Wnt signalling pathway in vascular endothelial cells by Wnt7b. This signalling cascade eventually results in the vascular remodelling. Thus, the new hypothesis is proposed, that the subpopulation of macrophages, modulating Wnt-signalling is located along the tumour vasculature to regulate endothelial cells proliferation and apoptosis [5].

## Conclusions

**Figure 11:**
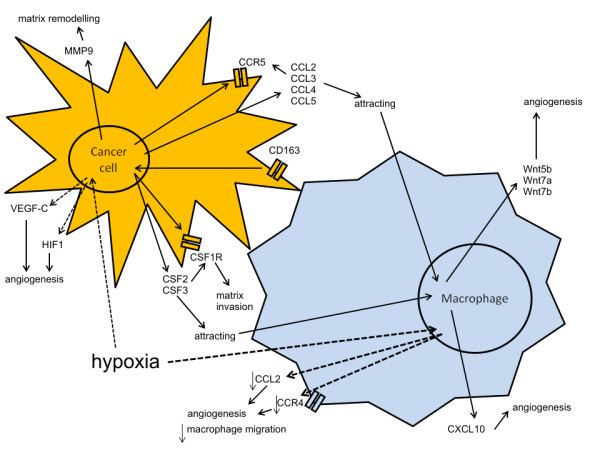
**Scheme of possible interactions between cancer cells and macrophages**. The scheme of possible interactions between the up/down-regulated genes in canine mammary cancer cell lines and macrophages. Abbreviations: CCL2, C-C motif chemokine 2; CCL3, C-C motif chemokine 3; CCL4, C-C motif chemokine 4; CCL5, C-C motif chemokine 5; CCR4, C-C chemokine receptor 4; CCR5, C-C chemokine receptor 5; CSF2 & CSF3, Colony stimulating factor 2 & 3; CXCL10, C-X-C motif chemokine 10; HIF1, Hypoxia inducible factor 1; MMP9, Matrix metalloproteinase-9.

Based on the results of hereby study and the available literature we conclude that the presence of macrophages in the cancer environment induces expression of macrophage-specific antigens in cancer cells. It may be caused by macrophage-cancer cell fusion or may be induced by chemokines. This macrophage-specific gene expression induces production of the pro-inflammatory mediators by cancer cells, that stimulate not only further monocytes recruitment from blood vessels and their differentiation into adult macrophages, but also cancer migration and angiogenesis (Figure 11). Moreover, we have showed that the presence of macrophages increases cancer cells migration and invasion. Thus, we suppose that gaining the macrophages phenotype by cancer cells constitutes one of the most important elements that increase their possibility to metastasize. Moreover, the 'cross-talk' between these cells leads to up-regulation of Wnt genes in macrophages increasing new vessels formation.

## Competing interests

The authors declare that they have no competing interests.

## Authors' contributions

MK research design, experimental design, FACS analyses and cell sorting, migration assay, manuscript and figures preparation, KP microarray analyses, real-time qPCR experiments, KM invasion assay, immunohistochemistry, MG confocal microscopy analyses, 3D culture, AM microarray analyses, TM manuscript preparation. All authors read and approved the final manuscript.
